# Transient Cannabinoid Receptor 2 Blockade during Immunization Heightens Intensity and Breadth of Antigen-specific Antibody Responses in Young and Aged mice

**DOI:** 10.1038/srep42584

**Published:** 2017-02-17

**Authors:** Emmanuel Dotsey, Irina Ushach, Egest Pone, Rie Nakajima, Algis Jasinskas, Donovan A. Argueta, Andrea Dillon, Nicholas DiPatrizio, Huw Davies, Albert Zlotnik, Peter D. Crompton, Philip L. Felgner

**Affiliations:** 1 Division of Infectious Diseases, Department of Medicine, University of California Irvine, Irvine, CA, USA; 2 Department of Pharmaceutical Sciences, University of California Irvine, Irvine, CA, USA; 3 Division of Biomedical Sciences, School of Medicine, University of California Riverside, Riverside, CA, USA; 4 Department of Physiology & Biophysics, University of California Irvine, Irvine CA, USA; 5 Malaria Infection Biology and Immunity Unit, Laboratory of Immunogenetics, NIAID, NIH, Rockville, MD, USA.

## Abstract

The hallmark of vaccines is their ability to prevent the spread of infectious pathogens and thereby serve as invaluable public health tool. Despite their medical relevance, there is a gap in our understanding of the physiological factors that mediate innate and adaptive immune response to vaccines. The endocannabinoid (eCB) system is a critical modulator of homeostasis in vertebrates. Our results indicate that macrophages and dendritic cells produce the endocannabinoid, 2-arachidonoyl-sn-glycerol (2-AG) upon antigen activation. We have also established that 2-AG levels are upregulated in the serum and in the lymph node of mice during vaccination. We hypothesized that the intrinsic release of eCBs from immune cells during activation by pathogenic antigens mitigate inflammation, but also suppress overall innate and adaptive immune response. Here we demonstrate, for the first time, that transient administration of the cannabinoid receptor 2 antagonist AM630 (10 mg/kg) or inverse agonist JTE907 (3 mg/kg) during immunization heightens the intensity and breadth of antigen-specific immune responses in young and aged mice through the upregulation of immunomodulatory genes in secondary lymphoid tissues.

Vaccines avert an estimated 2.5 million deaths each year^1^ and serve as an invaluable public health tool for combating the spread of infectious diseases. One drawback to the use of vaccines is the fact that they are only useful in individuals with functional immune system capable of mounting effective innate and adaptive immune response against foreign pathogens^2^. The elderly – one of the most rapidly expanding groups in the world^3^,^4^, are more vulnerable to infectious diseases due to the natural decline in immunity that occurs with age^5^. This phenomenon increases the frequency and severity of infectious diseases, and contributes to over 3% of pneumonia and influenza-related deaths in the elderly in the US alone^6^.

Found in all vertebrates, the endocannabinoid system is a ubiquitous signaling system consisting of membrane cannabinoid receptors 1 and 2 (CBR1 and CBR2) that are stimulated by Δ^9^-Tetrahydrocannabinol (Δ^9^-THC) - the psychoactive constituent of cannabis, and endogenous bioactive lipid ligands 2-archidonoylglycerol (2-AG) and anandamide (AEA), known as endocannabinoids (eCBs)^7^,^8^,^9^. ECBs are produced “*on demand*” from membrane phospholipid precursors and elicit their immune modulatory actions through downregulation of proinflammatory cytokines and TH17 cells^10^,^11^, modulation of CD4 + TH1/TH2 T cell responses^12^, upregulation of regulatory T-cells^13^,^14^, induction of immunosuppressive myeloid-derived suppressor cells^15^, inhibition of leukocyte proliferation and induction of apoptosis of effector cells^11^. Even though immune cells express both CB1R and CB2R, it is CB2R that has been implicated in immune regulation^16^,^17^. Among immune cells, B lymphocytes, Natural Killer (NK) cells and monocytes express the highest levels of CB2Rs - evidence that initially suggested that these receptors may play an important role in the function of immune cells^17^,^18^,^19^.

Our results indicate that bone marrow-derived dendritic cells and macrophages produce 2-AG upon activation by antigen. Previous studies using a methylated BSA (mBSA) antigen model of delayed type hypersensitivity (DTH) also revealed that activated T cells and B cells produce 2-AG which inhibit T-cell activation and proliferation^20^. In a study using the synthetic CBR agonist CP55 940, it was shown that the activation of CB2R during antigen presentation results in decreased MHC II molecules and CD4 + TH1/TH2 T Cell responses^12^. Additionally, Δ^9^-THC has been shown to impair the inflammatory response to influenza infection^21^. We therefore postulate that the *on-demand* engagement of eCB signaling during immunization serves as an inherent “braking system” to prevent excessive inflammation during immune activation, but also to set an inhibitory tone that suppress natural immune response to vaccination and may diminish immune responses, particularly in the elderly where age-associated immune decline affects both the innate and adaptive immune systems and decrease the capacity for antigen-specific immune response^22^,^23^. We further posit that it is paramount to suppress this eCB inhibitory tone for effective innate and adaptive immune responses to vaccination, more so in the elderly. This general dampening negative feedback control of eCBs has been well studied in other physiological systems^24^.

The idea of modulating immune response to vaccines and infection for a better outcome is not new. For example, monoclonal antibodies that target and reduce PD-1 and CTLA4 – negative modulators of adaptive immune response, have been successfully used in cancer immunotherapy^25^. Modulating the baseline immune status with drugs prior to vaccination has also been suggested as a promising strategy^2^ but so far, achieving this goal has been elusive. CB2R activation during antigen presentation leads to immunosuppression and anti-inflammation which dampens innate immune activation^17^. Here we investigate the effect of transient CBR2 blockade on immune response to vaccination in young and aged mice.

## Materials and Methods

### Pharmacological Agents

AM630, [6-iodo-2-methyl-1-[2-(4-morpholinyl)ethyl]-1H-indol-3-yl](4-methoxyphenyl)-methanone, and AM1241, (2-iodo-5-nitrophenyl)-(1-(1-methylpiperidin-2-ylmethyl)-1H-indol-3-yl)methanone were purchased from Cayman Chemical (Ann Arbor, Michigan USA)). JTE907, N-(benzo[1,3]dioxol-5-ylmethyl)-7-methoxy-2-oxo-8-pentyloxy-1,2-dihydroquinoline-3-carboxamide was purchased from Tocris Bio-Techne (Minneapolis, MN, USA). Lipopolysaccharides (LPS) from Escherichia coli were procured from Sigma (St. Louis, MO, USA.

### Antibodies and other Reagents

Brilliant Violet 421™ anti-mouse I-A/I-E Antibody, Brilliant Violet 510™ anti-mouse CD4 Antibody, Brilliant Violet 570™ anti-mouse/human CD45R/B220 antibody, Brilliant Violet 605™ anti-mouse IgM Antibody, Brilliant Violet 650™ anti-mouse CD25 antibody, Brilliant Violet 785™ anti-mouse CD8a antibody, PE/Cy7 Goat anti-mouse IgG (minimal x-reactivity) antibody, Alexa Fluor^®^ 488 anti-mouse/rat/human FOXP3 antibody, Alexa Fluor^®^ 647 anti-mouse IgD antibody, and APC/Cy7 anti-mouse CD138 (Syndecan-1) antibody were purchased from Biolegend. AnaSpec 7-AAD was procured from Fisher Scientific and Biotinylated Peanut Agglutinin (PNA), was purchased from Vector Labs. Biotinylated goat anti-mouse IgG, IgG1, IgA and IgM secondary Abs were purchased from Jackson ImmunoResearch Laboratories. For monitoring protein expression on arrays, monoclonal mouse anti-polyhistidine was procured from Sigma-Aldrich (St. Louis, MO, USA) and rat anti-hemagglutinin (HA; clone 3F10, anti-HA high affinity), from Roche (Pleasanton, CA) were used. Streptavidin-conjugated SureLight P3 was purchased from Columbia Biosciences (Frederick, MD).

### Protein purification and microarray fabrication

#### Protein purification

Immunogenic protein antigens FTT1269, FTT1329, FTT1483 (*Francisella tularensis*), WR148, WR101 (*Vaccinia virus*), BB_B19, BB_O34 (*Borrelia burgdorferi*), Rv3362 (*Mycobacterium tuberculosis*), and OTB1104 (*Orientia tsutsugamushi*) were purified as previously described^26^. Briefly, Protein-encoding T7-expression plasmids were used for protein expression in *E. coli* cells *in vivo*. Plasmids were transformed into *E. coli* strain BL21 Star (DE3) cells, and sequence verified. Master cell banks (MCBs) of a single clone that showed high protein expression *in vivo* were then deposited as glycerol stocks. Expression was evaluated by SDS PAGE and Western blot analysis using antibodies to the polyhistidine and hemagglutinin epitope tags engineered into the N- and C-termini of each protein, respectively^27^. Pilot expression experiments were first conducted in small cultures to determine expression levels and solubility characteristics of the protein. For this, cells scraped from frozen MCBs were inoculated into 5 ml LB broth containing 50 μg/ml kanamycin, and incubated at 37 °C with vigorous shaking (300 rpm) and aeration. Protein expression was induced with a final concentration of 0.1 mM IPTG when the culture reached an OD_600nm_ of 0.4–0.6, and incubation continued overnight until the culture reached stationary phase. Pelleted bacterial cells were lysed using BugBuster^®^ Protein Extraction Reagent (EMD Millipore, Billerica, MA) according to manufacturer’s instructions. After centrifugation, the pellet and supernatant fractions were examined by dot blot to determine the content of the protein. Scale-up cultures (1 L) were performed as above, except 1 ml starter cultures were inoculated in terrific broth (TB), containing 50 μg/ml kanamycin. Protein expression was induced with a final concentration of 0.1 mM IPTG when the culture reached an OD600 nm of 0.4–0.6 and incubation continued overnight until the culture reached stationary phase. Soluble proteins (FTT1269, FTT1329, FTT1483, WR101, BB_O34, Rv3362, and OTB1104) were recovered from the culture supernatant following centrifugation (16,000 g, 20 min, 4 °C), and purified by FPLC (ÄKTA, GE Healthcare, Pittsburgh, PA) on nickel columns as described^28^. Briefly, 5 ml HisTrap™ HP columns (GE Healthcare Life Sciences, Chalfont, UK) were equilibrated with phosphate buffered saline (PBS) containing 20 mM imidazole, pH 7.4 (binding buffer). Sample lysate was loaded and the column washed with binding buffer at a flow rate of 1 ml/min. Elution of protein was performed using a step gradient of 35% (175 mM imidazole) to 75% (375 mM imidazole) elution buffer at a flow rate of 1 ml/min. The elution buffer was then exchanged with PBS, pH 7.4, by dialysis. Insoluble proteins (WR148 and BB_B19) found as inclusion bodies (IBs) in the pellet fraction were extracted using BugBuster^®^. IB pellets were solubilized in 20 mM Tris-HCl, pH8.0, containing 7 M urea, 0.5 M NaCl, 5 mM imidazole, and 1 mM 2-mercaptoethanol, and protein purified by FPLC. Solubilized proteins were then bound to a HisTrap™ HP columns, and eluted with 75% (375 mM imidazole) elution buffer (7 M urea, 20 mM Tris-HCl, 0.5 M NaCl, 0.5 M imidazole, pH 8.0) at a flow rate of 1 ml/min. Eluted proteins were refolded using Slide-A Lyzer Dialysis Unit (Thermo Fisher Scientific, Waltham, MA) with a 10 kDa cut off, concentrated using centrifugal filter units with a 10 kDa cut off (Amicon Ultra-15, EMD Millipore) to ~1 mg/ml by Bradford assay, and stored in −80 °C. The average amount of protein recovered from a 1–2 L culture was 1–3 mg, with a purity of 80–90% as estimated by 4–12% gradient SDSPAGE. Purified *Borrelia burgdorferi* OspC-1 and OspC-K proteins were purchased commercially from GeneScript (Piscataway, NJ, USA).

#### Protein microarray fabrication and hybridization

All eleven constituent pathogenic proteins in vaccine were printed onto nitrocellulose-coated glass slides (Grace Bio-Labs), using an OmniGrid 100 microarray printer (Genomic Solutions) at a concentration of 1 mg/ml (1 ng protein/spot). Serum samples from vaccinated mice were diluted 1:100 in protein array blocking buffer (GVS, Sanford, ME, USA) supplemented with *E. coli* lysate (GenScript, Piscataway, NJ, USA) to a final concentration of 10 mg/mL, and preincubated at room temperature (RT) for 30 min. Concurrently, arrays were rehydrated in blocking buffer (without lysate) for 30 min. Blocking buffer was removed, and arrays were probed with preincubated serum samples using sealed chambers to ensure no cross-contamination of sample between pads. Following overnight incubation, slides were washed five times in washing buffer (10 mM Tris (pH 8.0), 150 mM NaCl), containing 0.05% Tween 20, and bound antibodies were detected by biotin SP-conjugated affini-pure goat anti-mouse IgG Fc fragment specific secondary antibody (Jackson ImmunoResearch, West Grove, PA), diluted 1/200 in blocking buffer. After 1 h at room temperature (RT), slides were washed three times, and bound antibodies were detected with streptavidin-conjugated SureLight P-3 tertiary reagent (Columbia Biosciences, Columbia, MD), diluted 1/200 in blocking buffer, for 1 hr at RT. Slides were then washed 5 times, air dried under brief centrifugation, and stored at 18 °C in a desiccator. The arrays were examined with a Perkin Elmer ScanArray Express HT confocal laser scanner at a wavelength of 670 nm and signal intensities were quantified using ProScanArray Express software (Perkin Elmer, Waltham, MA). All signal intensities were corrected for spot-specific background. Only signal intensities above a certain threshold signal defined by the mean + 2 SD of eight replicate spots of carrier buffer alone were considered positive, and used for the calculation of median signal intensities or percentages of bound proteins.

### Animals

Female C57BL/6 mice were used in our experiments. Young (8 weeks old) mice were purchased from Charles River. We procured 22 month old mice from the National Institute of Aging, NIH (Bethesda, MD, USA). Mice were housed (five animals per cage) under pathogen free conditions at the Gillespie Neuroscience Research Facility (GNRF) Vivarium. Food and water were available ad libitum and animals were kept under 12 hour light/12 hour dark cycle at a temperature 20–23 °C and humidity of 50–60%. All animal experiments were conducted under protocols approved by the University of California, Irvine, Institutional Animal Care and Use Committee (IACUC).

### Immunization and Drug administration

We developed a custom vaccine consisting of 11 purified recombinant immunogenic protein antigens from 5 medically important pathogens (2 μg/antigen) in 22 μL of PBS and formulated with equal volume of aluminum hydroxide gel (alum) as adjuvant. The formulation was mixed thoroughly by gentle pipetting. At day 0, mice were treated with established effect doses of cannabinoid receptor modulators 10 mg/kg AM630 (antagonist)^29^,^30^, 10 mg/kg JTE907 (inverse agonist)^31^,^32^, 3 mg/kg AM1241 (agonist)^33^,^34^ or vehicle (10% DMSO, 10% Tween-80, 80% normal saline) 30 minutes prior to immunization with 44 μL of vaccine/alum formulation into gastrocnemius muscle of hind leg, given vaccine boost at day 14 with experimental endpoint at day 28. Drugs were delivered by intraperitoneal (i.p.) route and continued till day 4 post-vaccination. Serum samples were collected from mice via saphenous vein at day 0, 7, 14, 21, and day 28 post-vaccination. At the experimental end-point at day 28, mice were sacrificed and spleens and lymph nodes harvested. For experiments to evaluate changes in endocannabinoid levels during vaccine-associated innate immune activation, animals were vaccinated while being treated with 10 mg AM630 or vehicle for 0, 2, or 4 days post vaccination.

### *In vitro* cell culture assays

Bone marrow-derived dendritic cells (BMDCs) and macrophages (BMM) were generated from 8-week-old C57BL/6 mice and activated with LPS as previously reported^35^,^36^ with minor modifications. Briefly, single cell suspension of bone marrow progenitor cells was prepared from mouse femurs. Cells were plated in 100-mm Corning^®^ culture dishes (10 ml/dish) plates and cultured at a density of 5 × 10^6^ cells/plate until fully differentiated. Cells were differentiated in complete RPMI 1640 Glutamax medium (Life Technologies) containing 10% heat inactivated fetal bovine serum (FBS) (Invitrogen), 100 μg/mL penicillin and 100 U/mL streptomycin (Invitrogen), and 30 ng/mL granulocyte-macrophage colony-stimulating factor (GM-CSF) (Biolegend) for BMDCs or 30 ng/mL macrophage colony-stimulating factor (Biolegend) for BMMs,. Culture media is gently replaced with fresh media every 3 days. After differentiation (7 days), cells were activated with 0.1 μg/mL LPS. After 24 hrs, cells were washed twice with PBS and harvested by gentle craping into 1 mL of ice cold PBS. Cells were then gently re-suspended in PBS and 20 μL of each sample was collected, counted and used for normalization. The samples were recovered by quick centrifugation and lipid analysis performed as described.

### Cell preparation and flow cytometry

Splenocytes and lymph node cells were harvested from vaccinated animals at the end-point experiment and single-cell suspensions prepared using a 70-mm cell strainer. Inguinal Lymph node cells were directly resuspended in complete RPMI 1640 medium after washing in PBS and centrifugation at 1500 rpm for 5 minutes. Splenocytes were resuspended in ACK Lysis Buffer (Lonza) to lyse RBCs followed by quenching with complete RPMI 1640. Cells were stained with the following antibodies showing optimized final concentration: Brilliant Violet 421™ anti-mouse I-A/I-E antibody (1 μg/mL), Brilliant Violet 510™ anti-mouse CD4 antibody (4 μg/mL), Brilliant Violet 570™ anti-mouse/human CD45R/B220 antibody (2 μg/mL), Brilliant Violet 605™ anti-mouse IgM antibody (4 μg/mL), Brilliant Violet 650™ anti-mouse CD25 antibody (4 μg/mL), Brilliant Violet 785™ anti-mouse CD8a antibody (4 μg/mL), Alexa Fluor^®^ 488 anti-mouse/rat/human FOXP3 antibody (1.2 μg/mL), Alexa Fluor^®^ 647 anti-mouse IgD antibody (10 μg/mL), and APC/Cy7 anti-mouse CD138 (syndecan-1) antibody (4 μg/mL), 7-AAD (20 μg/mL) and PE-Peanut Agglutinin (PNA) (4 μg/mL). Stained cells were then analyzed on a NovoCyte 3000 flow cytometer.

### Quantitative real-time PCR analysis

Quantitative real-time PCR (qRT-PCR) data was generated with a Lightcycler 480 (Roche). cDNA was obtained from total RNA extracted from mouse activated inguinal lymph node tissues using TRIzol (Invitrogen, Carlsbad, CA) and subsequently purified using Qiagen’s RNAesy columns and DNase digest (Qiagen). Gene-specific primers and corresponding Universal Probes were used to quantify indicated gene transcripts. Following gene-specific primers (5′ > 3′) were used to quantify indicated gene transcripts: CCR7: fwd-ctccttgtcattttccaggtg and rev-tggtattctcgccgatgtagt; CXCR5: fwd-gaatgacgacagaggttcctg and rev-gcccaggttggcttcttat; L-Selectin: fwd-tggcaaggcggttaaaaa and rev-aaaactgcagcagactgtgg; MHCII: fwd-cgccctagagagccagaaa and rev-ttgctggtacaggaagtaagc; SWAP70: fwd-ggagcgggagaagctgat and rev-tccagttcggaggacttctg; Connexin43: fwd-acgacaacctggagcaagat and rev-tgaggaagacagtggctatgc; IRF8 fwd-ccggcaagcaggattaca and rev-gctttgtctccctctttaaacttc; BCL-6: fwd-gaactgtatgcagattccagtca and rev-actgtcctcttgtaatccttcca; MCL1: fwd-ggtatttaagctagggtcatttgaa and rev-tgcagccctgactaaaggtc; E2A: fwd-ttccattttaacgtccatacagtc and rev-tgcagaaacacgcttttcata; EBF1: fwd-ccaactcaccctatgccatt and rev-ggggaggcttgtagatgagg; SPI-B: fwd-tctgaaccaccatgcttgc and rev-ctcaggccttggagacactc; BACH2: fwd-cagtgagtcgtgtcctgtgc and rev-ttcctgggaaggtctgtgat; MYC: fwd-gtgcctctcttttaaccagca and rev-tccttggctagcattaaagatattg; MGLL: fwd-cttcctcctgggccactc and rev-aggtgaaatcaggaccatgc; DAGLA: fwd-gagcaccaagcccaaatg and rev-agctccgacttggggatac; DGLB: fwd-aggattggtggcgactgt and rev-tggtcaccttccactgcat; ABHD6: fwd ttcttcaccccagcatcg and rev-caaacatgttaaccacatcgaga; ABHD12: fwd-ggatgatgtgactattggagtctg and rev-catctggtccttcccttgg; FAAH-1: fwd-gcaggtgggctgttcagt and rev-ccaagcagggatccacaa; CES1: fwd-agattcagtgaccgtctttgg and rev-tggccagaggagataagacaa; CES2: fwd-tttgtgggacatgaatttgg and rev-aattggcccagtacttcatca; GAPDH: fwd-tccactcatggcaaattcaa and rev-tttgatgttagtggggtctcg.

### Lipid Extraction

Blood was harvested from vaccinated mice by cardiac puncture and placed into tubes coated with EDTA dipotassium salt (Sarstedt, Nümbrecht, Germany). Blood was separated by centrifugation at 2000 g for 5 minutes at 20 °C and serum collected and stored at −80 °C. Inguinal, parotid, axillary, accessory axillary and submandibular lymph nodes were harvested and stored at −80 °C. Bone marrow dendritic cells and macrophages were harvested by gentle scrapping after rinsing twice with cold PBS. Frozen tissue or cells were homogenized in 1.0 mL of methanol solution containing the internal standard, [^2^H_5_] 2-AG and [^2^H_4_]-AEA (Cayman Chemical, Ann Arbor, MI, USA). Lipids were extracted with chloroform (2 mL) and washed with water (1 mL). Lipids were similarly extracted from serum samples, with the exception of a 0.9% saline wash replacing water (0.1 mL serum at the expense of saline). Organic phases were collected and separated by open-bed silica gel column chromatography as previously described (DiPatrizio *et al*., 2011, PNAS). Eluate was gently dried under N_2_ stream (99.998% pure) and resuspended in 0.1 mL of methanol:chloroform (9:1), with 1 μL injection for ultra-performance liquid chromatography/tandem mass spectrometry (UPLC/MS/MS) analysis.

### Lipid Analysis

Lipids were analyzed using a Waters Acquity I-Class Ultra Performance Liquid Chromatography system coupled to a Waters TQS-micro Triple Quadrupole Mass Spectrometer. Lipids were separated using an Acquity UPLC BEH C18 column (50 × 2.1 mm; i.d. 1.7 μm), eluted by a gradient of methanol (0.25% acetic acid, 5 mM ammonium acetate) in water (0.25% acetic acid, 5 mM ammonium acetate) (from 80 to 100% methanol in 2.5 min, 100% 2.5–3.0 min, 100–80% 3.0–3.1 min) at a flow rate of 0.4 mL/min. Column temperature was kept at 40 °C and samples were maintained in the sample manager at 10 °C. Argon was used as collision gas (99.998% pure). 2-AG, AEA, OEA, docosahexaenoyl glycerol (DHAG), docosahexaenoyl ethanolamide (DHEA), [^2^H_5_] 2-AG, [^2^H_4_] AEA, and [^2^H_4_] OEA were identified in the positive ionization mode based on their retention times and MS^2^ properties using authentic standards (Cayman Chemical; Ann Arbor, MI, USA) as references. Lipids were quantified using a stable isotope dilution method detecting protonated adducts of the molecular ions [M + H]^+^ in the multiple reaction monitoring (MRM) mode. Extracted ion chromatograms were used to quantify 2-AG (*m/z* = 379.2 > 287.26), AEA (*m/z* = 348.3 > 62.04), OEA (*m/z* = 326.3 > 62.08), DHAG (*m/z* = 403.3 > 311.19), DHEA (*m/z* = 372.3 > 91.02), and [^2^H_5_] 2-AG (*m/z* = 384.2 > 93.4), [^2^H_4_] AEA (*m/z* = 352.3 > 66.11) and [^2^H_4_] OEA (*m/z* = 330.3 > 66.05), which were used as internal standards. Lower limit of quantitation (LLOQ; signal-to-noise ratio of greater than 10) of analytes using our optimized UPLC/MS/MS methods were as follows (pmol): 2-AG, 0.5; AEA, 0.008; OEA, 0.06; DHAG, 0.5; DHEA, 0.008. Each analyte yielded the following percentage (%) of recovery between measured and actual value under our conditions (10 pmol; n = 3): 2-AG, 93.8; AEA, 103.4; OEA, 104.4; DHAG, 89.2; DHEA, 90.3.

### Statistical analysis

All statistical analyses were performed with Prism version 6.0 (GraphPad Software Inc., La Jolla, CA). Results are expressed as mean ± S.E.M. Statistical significance was assessed by Mann-Whitney test or Kruskal–Wallis one-way ANOVA test on ranks followed by Dunn’s post hoc analysis for multiple comparisons.

## Results

### 2-AG levels are upregulated in activated macrophages and dendritic cells and in serum and lymph nodes of vaccinated mice

**Figure 1:**
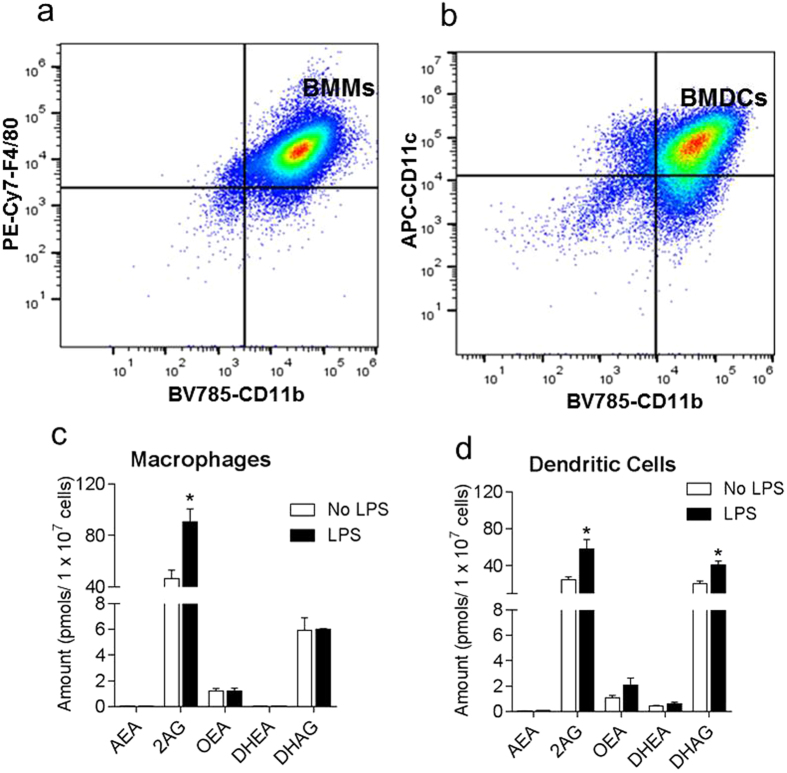
Flow cytometry and lipid analysis of Bone marrow derived macrophages (BMMs) and dendritic cells (BMDCs). (**a**) Purity of BMMs were analyzed using anti-F4/80-PE-Cy7 and anti-CD11b-BV785 antibodies. Matured and fully differentiated BMMs are double positive for F4/80 and CD11b (top right quadrant). (**b**) BMDCs were stained with anti-CD11c-APC and anti-CD11b-BV785 antibodies. Matured and fully differentiated BMDCs are double positive for CD11c and CD11b (top right quadrant). (**c**,**d**) Lipid analysis of BMMs and BMDCs activated overnight with 0.1 μg/mL LPS. Bar graphs are expressed as mean lipid amount ± SEM (n = 3). Statistical differences were determined by nonparametric Mann-Whitney U test, ^∗^P < 0.05, compared to No LPS control.

We began our studies of the role of the CBR system in immune response to vaccination by first testing the hypothesis that antigen presenting cells (APCs) produce bioactive lipids during activation that suppress innate immune activation. To test this hypothesis we generated bone marrow DCs (BMDCs) and macrophages (BMMs) from C57BL/6 mice. Prior to lipid analysis, we verified the purity of differentiated BMDCs and BMMs by flow cytometry using anti-F4/80-PE-Cy7, anti-CD11b-BV785 and anti-CD11c-APC antibodies^37^,^38^. Matured and fully differentiated BMMs were double positive for F4/80 and CD11b (Fig. 1a, top right quadrant) whereas matured BMDCs were double positive for CD11c and CD11b (Fig 1b, top right quadrant).

Mature BMDCs and macrophages were activated overnight with 0.1 μg/mL LPS as previously described^35^,^36^. We then performed lipidomic analysis to determine changes in the levels of anti-inflammatory bioactive lipid profile in cells post LPS activation. We evaluated levels of eCBs (2-AG and AEA), and the anti-inflammatory bioactive lipids N-Oleoylethanolamine (OEA), docosahexaenoic ethanolamide (DHEA) and docosahexaenoic glycerol (DHAG). The results revealed that levels of 2-AG were significantly increased in macrophages and DCs after LPS activation (Fig 1c,d). Levels of the anti-inflammatory fatty acid DHAG was also found to be elevated in DCs post activation but not in macrophages. No significant changes were detected in the levels of AEA, OEA or DHEA (Fig 1c,d) post-activation. The results suggest that in response to LPS, APCs produce eCBs to prevent excessive death-signaling. This observation led us to postulate that increased eCB signaling during innate immune activation mitigates inflammation, but also suppresses natural immune responses, and hence transient CBR2 antagonism during the innate immune activation stage of immune response to vaccination may enhance the overall antigen-specific adaptive immune response.

**Figure 2:**
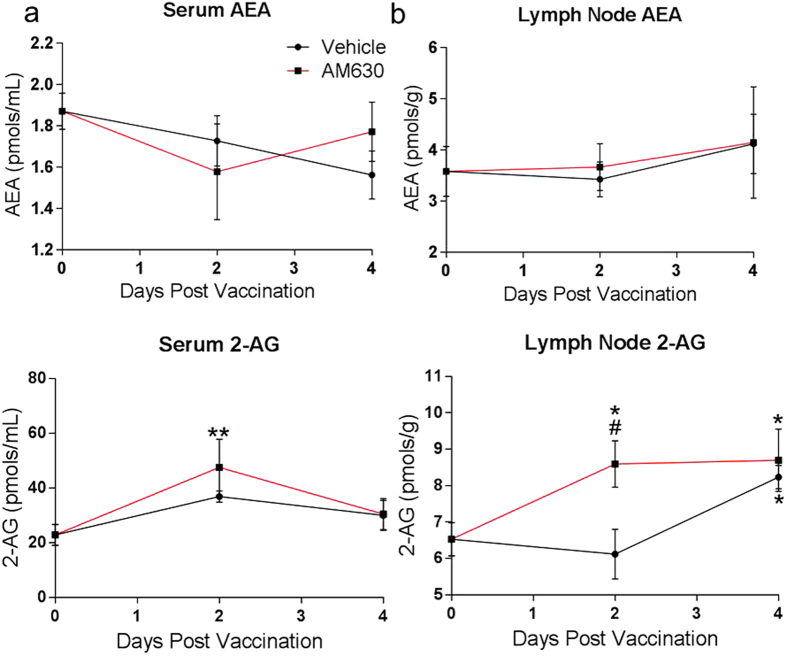
2-AG level is upregulated in serum and lymph nodes of vaccinated mice. To evaluate the levels of endocannabinoids in serum and lymph nodes during vaccination in mice, animals were immunized with P-11 mixed-antigen vaccine formulated in alum. Groups of 8 weeks old mice (n = 4) were treated with either 10 mg/kg AM630 or vehicle for 0, 2, or 4 days post vaccination. At each time point, mice were sacrificed and serum and lymph node tissues are harvested. Lipid analysis was then performed on sera and lymph nodes tissues to evaluate changes in AEA and 2-AG levels from days 0–4 post-vaccination. (**a**,**b**) AEA levels were not significantly altered in serum or lymph nodes of AM630 or vehicle treated mice at days 2 or 4 post-vaccination. (**c**,**d**) 2-AG levels are upregulated in serum and lymph nodes of AM630 and Vehicle treated mice during vaccination, but levels in the lymph nodes of AM630 treated mice are significantly higher at day 2 post-vaccination. Results are expressed as mean ± SD; n = 4/group. Statistical comparison of means was conducted by Kruskal-Wallis test, followed by Dunn’s multiple comparisons test, *p < 0.05 or **p < 0.01, compared with day 0 control mice. ^#^p < 0.05, compared with vehicle treated mice.

To further explore our initial findings, we examined changes in eCB levels in serum and activated lymph nodes of vaccinated mice during the innate immune activation stage - period preceding germinal center formation (days 0–4 post vaccination). Since 2-AG production in APCs in response to antigen activation (Fig. 1) likely serves a protective role to dampen excessive inflammation, we examined the question whether levels of 2-AG in serum and lymph nodes of vaccinated animals also increase during the innate immune activation stage of immunization – period preceding the establishment of the germinal center in activated lymph nodes. To answer this question, we vaccinated 8 week old mice (n = 5) with a custom alum-formulated vaccine (termed P-11 vaccine) consisting of 11 purified protein antigens from 5 medically important pathogens (Table 1). At day 0, mice were given an established effective dose of cannabinoid receptor antagonist AM630 (10 mg/kg)^29^,^30^, or vehicle thirty minutes before vaccination. Drug treatment was repeated at days 2 and 4 post vaccination. We selected time-points based on the fact that T-cell and B-cell differentiation activities in activated lymph nodes occur in phases, namely the early initiation phase (days 0–2) comprising of the period of B cell and T cell activation, the late initiation phase (days 3–4) which is characterized by T-cell and B-cell migration out of the interfollicular region of lymph node and the appearance of the early GC [36]. At each time point, mice were sacrificed and serum and activated inguinal lymph node tissues collected for lipid analysis to determine eCBs levels. As shown in Fig. 2, serum 2-AG levels in AM630 treated mice were significantly upregulated at day 2 but not at day 4 compared to baseline 2-AG levels at day 0. AM630 administration did not result in a significant increase in serum 2-AG compared to vehicle treated control at days 2 and 4 post vaccination (Fig. 2c). In the lymph node, CBR2 blockade resulted in a robust increase in 2-AG levels at days 2 and 4 post-vaccination (Fig. 2d). The 2-AG increase in lymph nodes of AM630-treated mice at day 2 was found to be significantly higher than baseline amounts at day 0 and also higher than levels in vehicle-treated mice at day 2 (Fig. 2d). On the other hand, in vehicle treated mice, 2-AG levels in the lymph nodes remained unchanged until after day 2 when it commenced to increase above baseline and reaching levels of AM630-treated mice by day 4 (Fig. 2d). On the contrary, we did not observe any significant changes in AEA levels in the serum or in the lymph node of AM630 treated mice compared to Vehicle controls.

### Pharmacological CBR2 blockade during early immunization in mice, upregulates expression of immunomodulatory and endocannabinoid-associated genes in activated lymph nodes

**Figure 3:**
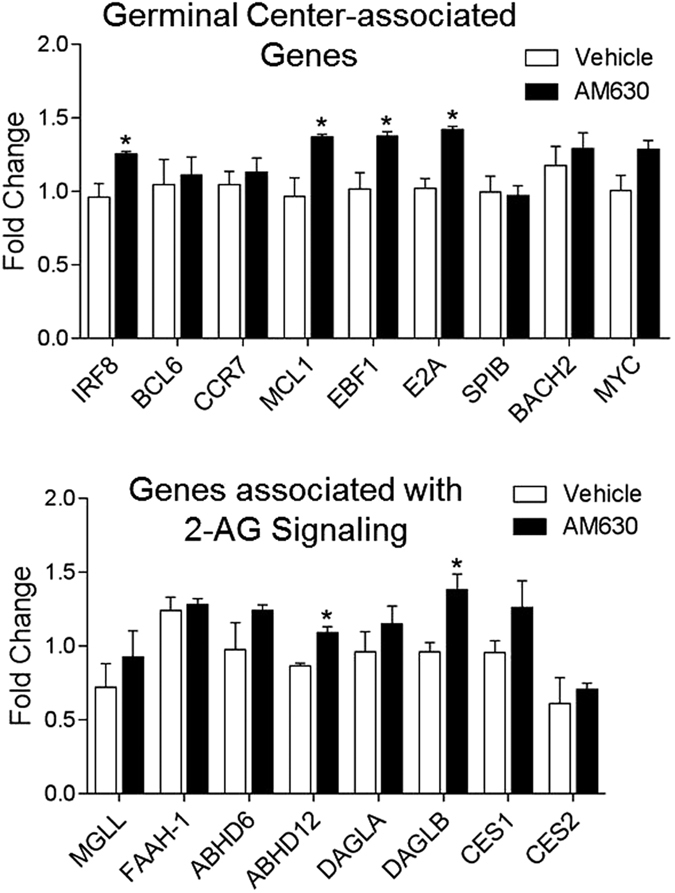
CBR2 antagonism during immunization upregulates specific Immunomodulatory genes and genes associated with endocannabinoid signaling in activated inguinal lymph nodes. Lymph nodes from Immunized 8 week old mice treated with 10 mg/kg AM630 or Vehicle from days 0–4 post-immunization were harvested at day 5 and analyzed by qPCR. Bar graphs derived from relative gene expression levels normalized to GAPDH and expressed as mean fold change over day 0 values ± SEM (n = 4), ^∗^P < 0.05, compared with vehicle, Statistical differences were determined by nonparametric Mann-Whitney U test.

We examined the effect of transient CBR2 blockade during vaccination on the expression of immunomodulatory genes which have been established to have a functional requirement for the progression of the germinal center (GC) reaction and B cell differentiation in both the GC and medullary chords of activated lymph nodes^39^ after immunization. To determine whether the increase in 2-AG in the lymph nodes following immunization is due to downregulation of constitutive 2-AG degradation or increase in biosynthesis, we evaluated changes in expression of 2-AG biosynthetic and metabolizing genes. Mice were treated with 10 mg/kg AM630^29^,^30^, or vehicle from days 0–4 post-vaccination. On day 5 post-vaccination, activated inguinal lymph node tissues were harvested and genes analyzed by qPCR using specific primers. T-cell and B-cell differentiation activities in activated lymph nodes occur in phases, namely the early initiation phase (days 0–2) comprising of the period of B cell and T cell activation, the late initiation phase (days 3–4) which is characterized by T-cell and B-cell migration out of the interfollicular region of lymph node and the appearance of the early GC^39^. Expression of GC-associated genes were analyzed at day 5 because it marks the beginning of proliferation and establishment phase of GC in activated lymph node^39^. The immunomodulatory genes analyzed are interferon regulatory factor 8 (*IRF8*), B cell lymphoma 6 (*BCL-6*), myeloid cell leukaemia 1 (*MCL-1*), *E2A*, early B cell factor 1 (*EBF1*), *MYC, SPIB, BACH2*^39^ and *CCR7*^40^. For 2-AG biosynthetic genes we evaluated *DAGLA and DAGLB* – genes that respectively encode the 2-AG synthesizing enzymes diacylglycerol lipase alpha (DGLα) and diacylglycerol Lipase β (DAGLβ)^41^,^42^. The 2-AG hydrolysing enzymes evaluated are monoacylglycerol lipase (MGL)^43^, alpha/beta-hydrolase domain containing 6 (ABHD6), alpha/beta-hydrolase domain containing 12 (ABHD12), Fatty Acid Amide Hydrolase 1 (FAAH-1), Carboxylesterase 1 (CES1) and Carboxylesterase 2 (CES2)^41^. The results indicate that out of the 9 immunomodulatory genes analyzed, transient CBR2 blockade during immunization led to the upregulation of 4 genes, namely *IRF8, MCL1, EBF1* and *E2A* (Fig. 3a) in activated lymph node tissues. Furthermore we observed upregulation of the endocannabinoid-associated genes *DAGLB* and *ABHD12* in activated lymph nodes but no significant changes in *DAGLA, ABHD6, FAAH-1, MGL, CES1* or *CES2* (Fig. 3b).

### Selective CBR2 modulation during vaccination heightens the intensity and breadth of antigen-specific antibody responses, and upregulates expression of immunomodulatory genes in the spleen

**Figure 4:**
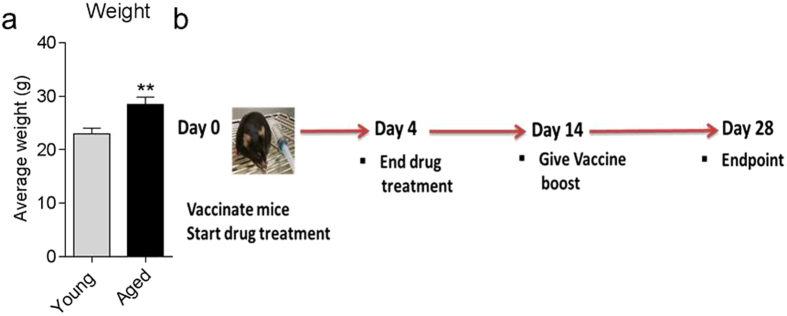
Weight of mice and immunization model. (**a**) Average weight of young (8 weeks old) and aged mice (20-month old) (**b**) P-11 vaccine immunization model. Mice were treated with CBR2 modulators 30 minutes prior to immunization with P-11 vaccine at day 0 and continued till day 4 post immunization. Animals were given a vaccine boost at day 14 with experimental endpoint at day 28. Statistical differences were determined by nonparametric Mann-Whitney U test, **p < 0.01, compared with young mice.

**Figure 5:**
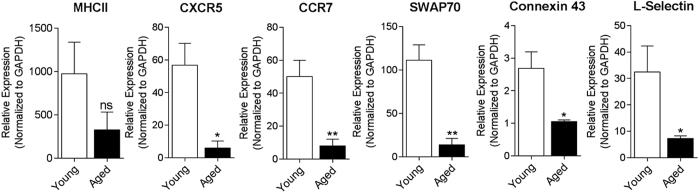
Aged mice exhibit diminished level of expression of immunomodulatory genes in the spleen in during immunization. Young and aged (20 month old) mice were immunized with P-11 vaccine at day 0, given a vaccine boost at day 14. At experimental endpoint at day 28, spleens were harvested and qPCR was performed on splenocytes using specific primers to determine the levels of immunomodulatory genes MHC II, CXCR5, CCR7, SWAP70, Connexin43 and L-Selectin. Gene expression levels were normalized to GAPDH. Results are expressed as mean ± SEM; n = 6–8/group. Statistical analysis conducted by nonparametric Mann-Whitney U test, *p < 0.05 or **p < 0.01, compared with young mice.

Innate immune activation is critical for effective immune responses, and precedes the establishment of the GC in secondary lymphoid tissues at day 4 post vaccination^44^. It has been established that natural immune response to vaccination diminishes with age^2^,^45^,^46^ and during the incidence of obesity^47^,^48^. Even though the average weight of aged mice 28.45 ± 1.38 g (mean ± SEM, n = 10) was significantly higher than that of young mice 22.90 ± 1.09 g (mean ± SEM, n = 10) (Fig. 4), both groups were not obese since the reported weight of obese C57BL/6 ranges between 47–54 g^49^. To test the hypothesis that CBR2 blockade during vaccination can lead to enhanced immune response, we first vaccinated aged mice with our custom P-11 vaccine (Table 1) using our vaccination model (Fig. 4b). At endpoint of vaccination at day 28, cDNAs were prepared from splenocytes and gene transcript levels of six immunomodulatory genes, MHC II^12^, CXCR5^40^, CXCR7^50^,^51^, SWAP70^52^,^53^, Connexin43^54^, and L-Selectin^55^ were determined by qPCR. Our results revealed that aged mice express diminished levels of all six genes we analyzed during immunization (Fig. 5). The results is consistent with previous studies that established that natural immune response wanes with aging^2^,^45^,^46^. We then evaluated the effect of transient CBR2 blockage on immune response to vaccination in aged mice compared to their younger cohorts by analyzing antigen-specific antibody responses and also levels of expression of immunomodulatory genes that we determined to have diminished expression in aged mice compared to young mice. To do this, mice were immunized with P-11 vaccine using our vaccination model (Fig. 4b) while being treated with established effective doses of CBR2 modulators; 10 mg/kg AM630 (antagonist)^29^,^30^, 3 mg/kg JTE907 (inverse agonist)^31^,^32^, 3 mg/kg AM1241 (agonist)^33^,^34^ or vehicle (10% DMSO, 10% Tween-80, 80% normal saline). Drugs were administered from days 0 to 4.

**Figure 6:**
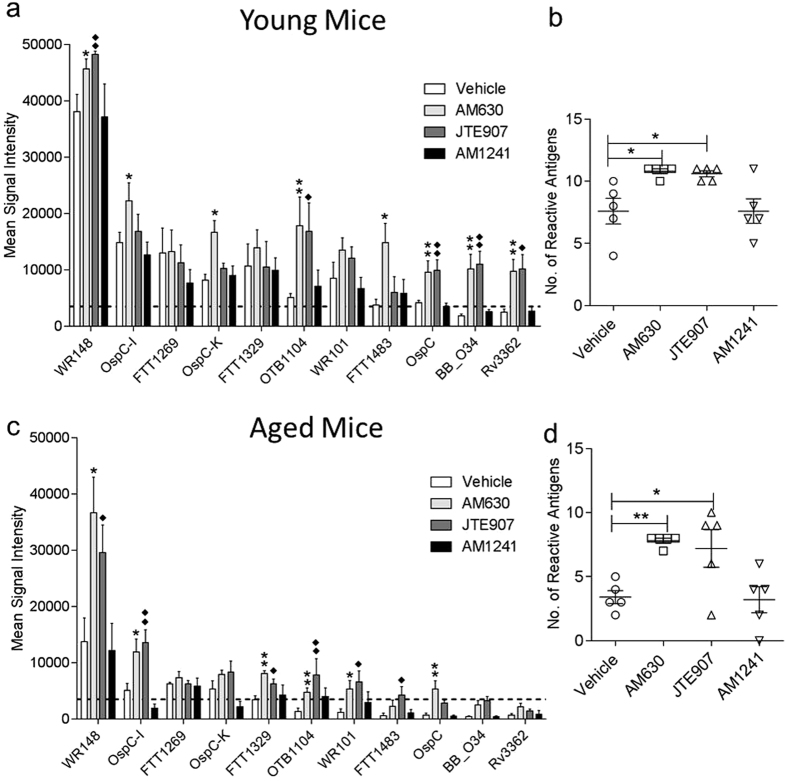
Transient CBR2 blockade during vaccination increases the intensity and breadth antigen-specific antibody responses in young and aged mice. 8 weeks old and 20 month old mice were immunized with P-11 mixed- antigen vaccine. Animals were vaccinated at day 0 and given a boost at day 14 with endpoint at day 28. Serum collected at day 28 post-vaccination was probed on a custom microarray printed with constituent proteins of P-11 vaccine. (**a**,**c**) Bar graphs representing mean signal intensities of serum antigen-specific IgG antibodies (**b**,**d**) Scatter plots representing number of reactive antigen-specific responses. Bar graphs and scatter plots expressed as mean ± SEM (n = 5). Statistical analysis performed by nonparametric Mann-Whitney U test, *p < 0.05 or **p < 0.01, all samples compared to vehicle control.

**Figure 7:**
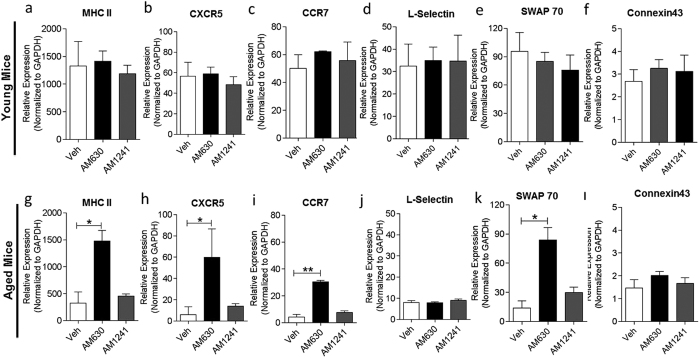
The effect of CB2R modulation on the expression of immunomodulatory genes in young and aged mice. To evaluate the effect of CBR2 modulation on expression of immunomodulatory genes in vaccinated young and aged mice, splenocytes from mice treated with either vehicle, 10 mg/kg AM630 or 3 mg/kg AM1241 from days 0–4 post-immunization were harvested and single-cell suspension prepared at end point of vaccination at day 28 post-vaccination. QPCR was performed on splenocytes using specific primers to determine the levels of immunomodulatory genes (**a**,**g**) MHC II, (**b**,**h**) CXCR5, (**c**,**i**) CCR7, (**d**,**j**) L-Selectin, (**e**,**k**) SWAP70 and (**f**,**l**) Connexin43. Gene expression levels were normalized to GAPDH. Results are expressed as mean ± SEM; n = 6–8/group. Statistical analysis conducted by nonparametric Mann-Whitney U test, *p < 0.05 or **p < 0.01, all samples compared to vehicle control.

To evaluate antigen-specific responses in serum, we produced a custom microarray chip with printed constituent protein antigens of the P-11 vaccine. At the experimental endpoint at day 28, serum was collected from vaccinated animals and the intensity and breadth of antibody responses was analyzed on our custom chip. The results revealed that transient CBR2 blockade during the innate immune activation phase of immune responses to immunization, increases the mean signal intensity of antigen-specific antibody responses in young and aged mice compared to control mice (Fig. 6a,c). Furthermore, the breath of response, defined as the number of reactive antigen responses in serum, was also found to be increased in young and aged mice treated with the CBR2 antagonist or inverse agonist compared to vehicle treated controls (Fig. 6b,d). Upregulation of eCB signaling suppresses immune response^17^, so we anticipated that further treatment of mice with exogenous CBR2 agonist AM1241 will lead to complete abrogation of immune response, but contrary to expectation, we did not see any significant differences between vehicle and AM1241 in treated young and aged mice (Fig. 6). This observation suggests that CBR2s may already be saturated by upregulated endogenous 2-AG. We also analyzed spleen tissues from vaccinated young and aged mice treated with AM630 or AM1241 by qPCR to determine the effect of CB2R blockade on expression of the immunomodulatory genes MHC II^12^, CXCR5^40^, CCR7^50^, SWAP70^52^,^53^, Connexin43^54^ and L-Selectin^55^ which we previously demonstrated to be downregulated in aged mice. The results indicate that CBR2 blockade significantly upregulated MHC II, CXCR5, SWAP70 and CCR7 expression levels in aged mice but not in young mice (Fig. 7).

## Discussion

Since its discovery and subsequent elucidation as the master modulator of homeostasis in vertebrates, the endocannabinoid system has become a promising target for the development of drugs for the treatment of a myriad of diseases^56^,^57^. Here we evaluate an approach by which cannabinoid signaling in immune cells can be harnessed to enhance innate and adaptive immune response during vaccination in mice. We hypothesized that the eCBs produced by immune cells during vaccination serve to restrict the extent of innate immune activation, and by so doing, suppress overall immune response. We therefore posit that decreasing CBR2 signaling through transient antagonism or inverse agonism during vaccination will relieve eCB inhibitory effects and facilitate innate and adaptive immune responses. Our findings showing 2-AG production in mouse dendritic cells and macrophages upon LPS activation (Fig. 2) agrees with earlier studies that demonstrated increased 2-AG production in activated human DCs^58^. Recent studies also reported that naïve T cells and B cells constitutively expressed low levels of 2-AG that significantly increases upon antigen activation^20^. One interesting observation in all these studies, including ours, is that, the endocannabinoid that is consistently upregulated in APCs during activation is 2-AG, but not AEA. The structure of the hydrolyzing enzymes of these endocannabinoids may account for their differential upregulation in APCs during immune activation. We previously reported in studies using brain neurons that the 2-AG hydrolyzing enzyme MGL is sensitive to oxidative inactivation^59^. Even though MGL and FAAH are both serine hydrolases and possess a catalytic serine^60^, MGL has a mobile and flexible lid domain that regulate access to the active site of the enzyme^61^. The lid domain of MGL contain essential cysteines residues C201 and C208 that when oxidized, immobilizes the lid, rendering the enzyme inactive^59^. In the context of immune cells, it is unclear whether post-translational oxidative modification of MGL accounts for the upregulation of 2-AG in APCs during innate immune activation (Figs 1 and 2) and more studies are needed to shed light into this phenomenon.

The quality of an antigen-specific immune response depends on efficiency of the initial innate immune activation and differentiation events that ensue in the activated lymph node. Our results show that alleviating the immunosuppressive effects of eCBs during early stages of vaccination enhances the overall intensity and breadth of antigen-specific immune responses in young and aged mice. The results also suggest that the enhancement in immune response elicited by CBR2 blockade during immunization was due in part to the upregulation in the expression of the immunomodulatory genes Mcl-1, IRF8, E2A and EBF1 in the lymph node. These genes have previously been established to regulate B-cell lineage specification, commitment, and differentiation^62^,^63^,^64^, and also to play an instructive role in early B cell lymphopoiesis and GC B cell development^65^,^66^. We noticed that even though CBR2 blockade resulted in the upregulation of IRF8, a factor is known to contribute the induction of BCL6^39^, this increase in IRF8 by CBR2 blockade did not result in further increase BCL6 levels compared to vehicle treated control. This observation could be due the fact that the induction and expression of BCL6 in differentiating B-cells by IRF8 begins at the early initiation stage of GC formation (Day 2 post-vaccination) and continue through the proliferation and establishment phase which starts at Day 5 and end at Day 7 post-vaccination^39^. We analyzed levels of genes at Day 4 post vaccination which marks the time at which the GC is first established^39^. In the current study, we cannot fully answer the question whether upregulation of IRF8 by CBR2 blockade resulted in a further increase in BCL6 level since we did not examine BCL6 levels at all timepoints beyond Day 4 post-vaccination during which BCL6 is expressed and further B-cell differentiation occurs in the germinal center^39^.

To determine whether the increase in 2-AG in activated lymph nodes following immunization is due to downregulation of constitutive 2-AG degradation or increase in biosynthesis, we also evaluated changes in gene expression of 2-AG biosynthetic and metabolizing enzymes. The spatial and tissue distribution of *DAGLA* and *DAGLB* accounts for their physiologic function^41^,^42^,^67^. *DAGLA* is spatially expressed in the CNS to targets CBR1 on neurons, whereas *DAGL*B targets CB2R in peripheral tissues^67^. Our results depicting an upregulation of *DAGLB* in activated lymph nodes suggest that increased 2-AG biosynthesis by *DAGLB* may be responsible for the increase in 2-AG levels observed in activated lymph nodes during vaccination. Furthermore, analysis of 2-AG hydrolytic genes revealed an increase in expression of *ABHD12* - a recently discovered hydrolase reported to contribute to 2-AG hydrolysis in the brain^68^. While the functional role of ABHD12 in 2-AG metabolism in the CNS has been elucidated^69^, its role in 2-AG hydrolysis in peripheral tissues is poorly understood. It is important to note that of all the 2-AG metabolizing enzymes, MGL is the major player, accounting for approximately 85% of the total 2-AG hydrolysis while ABHD6, ABHD12 and FAAH-1 account for the remaining 15%^41^,^68^. Indeed, more studies are needed to shed light into the role of ABHD12 in 2-AG hydrolysis during immune activation. It is also noteworthy that hydrolysis of 2-AG does not terminate its anti-inflammatory effects, but rather, releases arachidonic acid that trigger a cascade of reactions through the cyclooxygenase-2 (COX-2) pathway leading to the production of prostaglandin (PG) analogs that have been elucidated as potent mediators of inflammation^41^,^70^.

The principal goal of this study is to evaluate the hypothesis that transient CBR2 blockade during immunization can heightened immune response. Natural immune response to vaccination has been shown to diminishes with age^2^,^45^,^46^, hence testing our hypothesis in young and aged mice allowed us to assess the ability of CBR2 blockers to restore the deficit in immune response found in aged mice compared to their younger cohorts. The results show that transient CBR2 blockade during immunization increases the intensity and breadth of antigen-specific antibody responses in young and aged mice compared to control mice (Fig. 6a,c). Upregulation of eCB signaling suppresses immune response^17^, so we anticipated that further treatment of mice with exogenous CBR2 agonist AM1241 will lead to complete abrogation of immune response, but contrary to expectation, we did not see any significant differences between vehicle and AM1241 treated young and aged mice (Fig. 6). Similarly, in a separate experiment using the CBR2 specific agonist HU-308 failed to augment immune response to vaccination (results not shown). This observation could be due to the fact that CBR2s may already be saturated by upregulated endogenous 2-AG.

We limited the duration of use of these agents to 4 days post vaccination, which marks the period of antigen activation and initiation of germinal center formation in secondary lymphoid tissues^39^. Indeed, the doses of AM630 (10 mg/kg) and JTE907 (10 mg/kg) and AM1241 (3 mg/kg) used in our investigations correspond to established effective doses^29^,^30^,^31^,^32^,^33^,^34^. Higher dose of AM630 (20 mg/kg) has been used with no detrimental side effects in mice^30^. In addition, prolonged oral administration of JTE907 (10 mg/kg) for 20 days to study the effect of CBR2 blockade on spontaneous pruritis-associated responses in a mouse model of atopic dermatitis showed that 10 mg/kg JTE907 administered for a prolonged period of 20 days effectively suppressed spontaneous pruritis-associated responses in this model without any adverse side effect^31^. While these studies may lend credence to the use of CBR2 blockers as enhancers of immune response to vaccination, it is important to note that eCB production during immunization serves a protective role by dampening excessive inflammation, hence further studies are needed to address the pressing question whether transient CBR2 blockage may precipitate unintended consequences such as toxic cytokine storm or breach in peripheral tolerance. Furthermore, it has been established that eCBs suppress excessive immune activation partly through the induction of apoptosis of effector cells^71^ – a process that can be hampered by CBR2 blockade during immune response. Further studies are needed to define the effect of CBR2 blockade during early vaccination on eCBs modulation of effector immune cells survival and function.

Endocannabinoid modulation of immune response is context-dependent. For example, 2-AG and CBR2s are upregulated in immune cells immediately after antigen activation^20^,^72^, but CBR2 signaling is substantially downregulated at the receptor level during B cell differentiation in the germinal center (GC), with lowest CBR2 expression reported in GC Centroblasts^72^. Eventually, mRNA and protein levels of CBR2s are restored in matured B cells exiting the GC as memory B^72^. Studies using CBR2 knockout have established the functional requirement of CBR2 signaling for memory B cell homing and retention of marginal zone B cells and for efficient T-independent Immune Responses^73^. Taken together, these results suggest that CBR2 signaling differentially modulate immune response during vaccination, by suppressing innate immune activation and GC B cell differentiation during early immune response, while facilitating migration and homing of memory B cell exiting the GC. Context-dependent CBR modulation has been shown in other systems^24^. Our model of CBR2 blockade during the first 4 days of vaccination does not overlap, and likely does not interfere with proliferation events and establishment of the matured GC from days 5–7^39^ post-vaccination. For the same reason, we also do not expect CBR2 blockade at the early onset of immunization to adversely affect antigen-specific B cell homing to the marginal zone of secondary lymphoid tissues which occurs later after differentiated memory B-cells exit the GC^73^. Furthermore, it has been reported that hematopoietic cells expressing CBR2 migrate in response to the endocannabinoid 2-arachidonoylglycerol^74^, hence further studies are needed to elucidate the effect of CBR2 antagonism on hematopoietic cell migration during early vaccination.

Our analysis of the critical immunomodulatory genes MHC II^12^, CXCR5^40^, CCR7^50^, SWAP70^52^,^53^, Connexin43^54^ and L-Selectin^55^ (Fig. 7) in the spleen of vaccinated animals revealed that CBR2 blockade did not significantly alter the levels of these genes in the spleen of young mice at day 28 post-vaccination. However, we observed that levels of MHC II, CXCR5, and CCR7 and SWAP70 were significantly upregulated in the spleen of aged mice treated with CBR2 blockers compared to control at the end of our vaccination model at day 28 post-vaccination. Collectively, these genes play a critical role in the orchestration of antigen processing and presentation (MHCII)^75^, direction of B cell migration to lymph nodes (CXCR5)^40^, coordination of primary immune response in secondary lymphoid tissues and maintenance of Th17 Immunity (CCR7)^76^,^77^, control of B cell homing, and the formation of splenic marginal zone (SWAP70)^52^,^53^. Indeed, while CBR2 blockade-driven increase of MHC II, CXCR5, CCR7 and SWAP70 in the spleen may contribute to the heightened immune response to immunization in aged mice, more studies are needed to expand our understanding of how CBR2 blockade generally enhance immune response in young and age mice. The results suggest that the upregulation of gene expression induced by CBR2 blockade during early immunization in secondary lymphoid tissues of aged mice may persist for at least 28 days post-immunization and may account for the enhanced immune response observed in treated animals. It is important to caution that slight differences exist in how eCBs modulate immune cells in mice compared to humans^17^, hence there is the possibility that the results that we demonstrate here in mice may differ in humans.

## Additional Information

**How to cite this article**: Dotsey, E. *et al*. Transient Cannabinoid Receptor 2 Blockade during Immunization Heightens Intensity and Breadth of Antigen-specific Antibody Responses in Young and Aged mice. *Sci. Rep.* **7**, 42584; doi: 10.1038/srep42584 (2017).

**Publisher's note:** Springer Nature remains neutral with regard to jurisdictional claims in published maps and institutional affiliations.

## Figures and Tables

**Table 1:** Constituent protein antigens, source pathogens, and amount in P-11 vaccine.

Pathogen	Antigen ID	Amount (μg)
*Francisella tularensis*	FTT1269	2
*Vaccinia virus*	WR148	2
*Borrelia burgdorferi*	OspC-1	2
*Borrelia burgdorferi*	OspC-K	2
*Orientia tsutsugamushi*	OTB1104	2
*Francisella tularensis*	FTT1483	2
*Francisella tularensis*	FTT1329	2
*Mycobacterium tuberculosis*	Rv3362	2
*Vaccinia virus*	WR101	2
*Borrelia burgdorferi*	OspC	2
*Borrelia burgdorferi*	BB_O34	2
